# Epidemiology of self-rated health in rural China: a population-based cross-sectional study

**DOI:** 10.1038/s41598-017-04381-6

**Published:** 2017-06-30

**Authors:** Fangfang Liu, Chaoting Zhang, Yongmei Liang, Qiuju Deng, Dong Hang, Yaqi Pan, Xiang Li, Zhonghu He, Mengfei Liu, Ying Liu, Jingjing Li, Tao Ning, Chuanhai Guo, Ruiping Xu, Lixin Zhang, Hong Cai, Yang Ke

**Affiliations:** 1 Key Laboratory of Carcinogenesis and Translational Research (Ministry of Education/Beijing), Laboratory of Genetics, Peking University Cancer Hospital & Institute, No. 52 Fucheng Road, Haidian District, Beijing, 100142 China; 2 Anyang Cancer Hospital, No. 1 Binhebei Rd, Anyang, Henan Province 455000 China

## Abstract

Self-rated health (SRH) has been shown to be a good predictor of mortality. Data on SRH and its associated factors in the Chinese general population are limited. This study aims to assess the epidemiology of SRH in rural Anyang, China. SRH (categorized as “healthy”, “fair” or “unhealthy”) was measured in a population-based study of 2,814 adults (including 697 couples) aged 25 to 69 who were recruited from rural Anyang in 2014. Of 2,814 subjects, 63.1% rated their health as “healthy”, whereas 28.1% and 8.8% rated their health as “fair” and “unhealthy”. Compared to males, females had a higher likelihood of reporting a better SRH. Health ratings declined with increasing age, unmarried status, lower education levels. Poor SRH was positively correlated with medical history as well as high levels of fasting plasma glucose and total cholesterol, but not with unhealthy lifestyle indicators including smoking, drinking, and obesity. High household income was predictive of better SRH in men but not in women. Among couples, a positive spousal SRH concordance was observed, although the strength of this concordance was low. These findings will be useful for formulation of appropriate strategies for improving risk perception and promoting general health in economically developing regions.

## Introduction

Self-rated health (SRH), which is a subjective perception of an individual’s health status, refers to a single-item health evaluation in which people are asked to assess their current health status on a five-point scale which ranges from very good to very poor^1, 2^. Many international studies have consistently demonstrated that SRH is a good predictor of mortality and is closely associated with morbidity and disability^2–4^. Moreover, SRH has been found to have good test-retest reliability^5^ and has been recommended for health monitoring by the World Health Organization and the European Union Commission^6^.

The prevalence and determinants of SRH have been extensively investigated mainly in economically developed countries. Medical health status and its subsequent functional outcomes are widely recognized as major determinants when people rate their health^7, 8^. However, the relationship between certain socioeconomic factors, health behaviors, clinical parameters and SRH varies depending on demographic and geographic factors^9–14^. In contrast with abundant data available from developed regions, studies in economically developing countries including China are not adequate and have generally been restricted to particular subpopulations, such as elderly people and floating population who refers to migrants without local household registration^11, 15, 16^. Furthermore, comprehensive assessments of the correlates of SRH among general Chinese populations are scarce. Most previous studies only evaluated the potential effects of interview data such as lifestyle characteristics on SRH ratings^11, 15, 16^. Other factors such as biochemical indicators, which may reflect subclinical physiologic conditions, have not been sufficiently investigated in existing studies carried out in China.

In addition, data concerning SRH spousal correlation is limited both in economically developed and developing regions. It has been suggested that good SRH is positively associated with spouses’ optimism^17^. In theory, environment and lifestyles shared by a couple could result in shared health risk^18^. However, factors which couples do not share such as genetic characteristics may cause spousal SRH discrepancy. The presence or absence of SRH correlation and the degree of SRH concordance in couples remains unclear.

To overcome these limitations and fill important research gaps, this population-based cross-sectional study seeks to (1) investigate the distribution of SRH and its determinants including socio-demographic characteristics, behavioral factors, physical status and clinical measurements in rural China, and (2) assess the correlation and concordance of spousal SRH in this population.

## Materials and Methods

### Study subjects

A population-based prospective cohort study of risk factors for esophageal cancer in rural Anyang, China, has been described elsewhere^19^. The current investigation utilized a subset including 5 of the 9 target villages which were cluster-sampled in the parent cohort study conducted in 2014. Eligibility criteria for enrolment were as follows: (1) permanent residency in the target villages; (2) age 25–69; (3) no prior diagnosis of cancer or mental illness; (4) no history of infection with HBV, HCV or HIV; (5) willingness to participate in the follow-up program and provide informed consent. All participants provided written informed consent, and the study was approved by the Institutional Review Board of the School of Oncology, Peking University. The methods were carried out in accordance with the approved guidelines.

### Questionnaire survey

A one-on-one computer-aided interview was administered in a private room by a trained interviewer, and information on SRH, demographic characteristics (age, gender and marital status), socioeconomic variables (number of family members living together, type of employment, education level, and household annual income), indicators of healthy or unhealthy lifestyle (cigarette smoking, alcohol drinking, and body mass index), and disease status (history of disease) were collected during these interviews. SRH was initially recorded in 5 levels (very good, good, fair, poor, and very poor), and then categorized into 3 groups which included “healthy” (very good and good), “fair” and “unhealthy” (poor and very poor) for statistical analyses. Annual household income was categorized as low, moderate and high on the basis of annual income of ≤10,000, 10,001–30,000 or >30,000 RMB. Cigarette smoking was defined as consuming one cigarette or more per day for ≥12 months, and alcohol consumption was defined as drinking Chinese liquor at least twice per week for ≥12 months. A former smoker or drinker was characterized as an “ever” user who had not smoked or consumed alcohol in the preceding 12 months. Body mass index (BMI) was calculated as weight/height^2^ (kg/m^2^) and categorized into 4 groups: underweight (BMI < 18.5), normal weight (18.5 ≤ BMI < 24.0), overweight (24.0 ≤ BMI < 28.0), and obesity (BMI ≥ 28.0)^20^. History of disease was referred to as any disease(s) diagnosed by medical doctors.

### Clinical measurement

Blood samples were obtained after overnight fasting. Clinical parameters were categorized as follows: fasting plasma glucose (FPG) (Normal: FPG < 6.1, borderline high: 6.1 ≤ FPG < 7.0, high: FPG ≥ 7.0 mmol/L), total cholesterol (TC) (Normal: TC < 5.2, borderline high: 5.2 ≤ TC < 6.2, high: TC ≥ 6.2 mmol/L), triglycerides (TG) (Normal: TG < 1.7, borderline high: 1.7 ≤ TG < 2.3, high: TG ≥ 2.3 mmol/L), high-density lipoprotein (HDL) (Normal: LDL < 3.4, borderline high: 3.4 ≤ LDL < 4.1, high: LDL ≥ 4.1 mmol/L) and low-density lipoprotein (LDL) (Normal: HDL ≥ 1.0, low: HDL < 1.0 mmol/L)^21, 22^. TC, TG, LDL and HDL were measured enzymatically on an automatic analyzer (Hitachi 7600–020, China) with reagents purchased from Sichuan Xin Cheng Bio-Tech Co., Ltd. FPG was measured on the fasting blood glucose meter (Yicheng 5D-2, China). All clinical measurements were performed by the clinical laboratory staff of Hua County Hospital, Anyang, China.

### Statistical analyses

The chi-square test was used to determine differences in self-rated health across model covariates. Due to the order of the outcome categories and satisfaction of the proportional odds assumption (measured by likelihood ratio tests and the Brant test), univariate and multivariate ordered logistic regressions adjusting for intracluster correlation (cluster variable: village) were conducted to estimate the relationships between explanatory variables and SRH. Potential risk factors that were statistically significant in univariate analyses, together with related variables which have been previously reported were included in multivariate models (Linear regression models were used to determine whether any variables in the multivariate models were highly collinear. In this study, all variance inflation factors were below 3.0 and therefore within the acceptable range). The odds ratios (ORs) and 95% confidence intervals (CIs) were calculated treating the group “healthy” as the reference group. Only a single odds ratio that described the odds of increasing by 1 category of the outcome for a 1-unit change in the explanatory variables was reported. For couple concordance analysis, Spearman’s rank test and weighted kappa statistics employing a linear set of weights (e.g., 1.0, 0.5, and 0.0) were used. The kappa statistics examine the level of agreement beyond what would happen by chance. The kappa value 0 indicates that the degree of agreement is no more than would be expected by chance, while the kappa value 1 indicates a perfect agreement.

Statistical analyses were performed using STATA version 12.0 software (STATA Corporation, College Station, TX, USA). *P* values less than 0.05 (two-sided) were considered to be significant.

## Results

### Participant characteristics

Of 3,457 eligible residents, 2,814 (81.4%) were enrolled. The main reason why the other 643 individuals, who were generally more likely to be younger men, did not participate was loss of contact due to employment outside of Anyang. The median age of these 2,814 participants was 49 (interquartile range, 43–60), with a female to male ratio of 1.6 (Table 1). Most of these subjects were married (94.2%), engaged in farming (73.2%), were lifetime non-smokers (74.5%) and lifetime non-drinkers (83.3%). The percentages of subjects had 1–2, 3–5, and ≥6 family members living together were 19.1%, 54.7%, and 26.3%, respectively. Almost half of the participants were illiterate or had a primary school level of education (48.5%). Subjects with low, moderate or high levels of household annual income each accounted for approximately one third of the study population. More than 60.0% of subjects were overweight or obese (64.4%). Fifteen percent had clinical disease currently or in the past. Proportions of subjects with borderline high/high levels of FPG, TC, TG, LDL and HDL were 7.0%, 29.3%, 30.1%, 13.4%, and 25.5% respectively.

**Table 1:** Socio-demographic characteristics and laboratory parameters by self-rated heath among 2,814 adults in rural China, 2014.

Variables^a^	Self-rated health				P values^b^
	Total, No. (%)	Healthy, No. (%)	Fair, No. (%)	Unhealthy, No. (%)	
***Total***	2,814 (100)	1,776 (63.11)	791 (28.11)	247(8.78)	
	***Demographic characteristics***				
***Age (years)***					<0.001
Median (IQR)	49 (43–60)	48 (41–57)	53 (46–63)	58 (49–65)	
25–40	496 (17.63)	403 (22.69)	82 (10.37)	11 (4.45)	
41–56	1,348 (47.90)	894 (50.34)	359 (45.39)	95 (38.46)	
57–69	970 (34.47)	479 (26.97)	350 (44.25)	141 (57.09)	
***Gender***					<0.001
Male	1,066 (37.88)	508 (28.60)	457 (57.77)	101 (40.89)	
Female	1,748 (62.12)	1,268 (71.40)	334 (42.23)	146 (59.11)	
***Marital status***					<0.001
Never married, divorced or widowed	161 (5.80)	75 (4.27)	63 (8.08)	23 (9.50)	
Married	2,616 (94.20)	1,680 (95.73)	717 (91.92)	219 (90.50)	
	***Socioeconomic characteristics***				
***Number of family members living together***					<0.001
1–2	437 (19.07)	212 (14.65)	166 (26.39)	59 (27.31)	
3–5	1,253 (54.67)	843 (58.26)	311 (49.44)	99 (45.83)	
≥6	602 (26.27)	392 (27.09)	152 (24.17)	58 (26.85)	
***Type of employment***					<0.001
Farming	2,034 (73.24)	1,304 (74.30)	533(68.33)	197 (81.40)	
Non-farming	743 (26.76)	451 (25.70)	247 (31.67)	45 (18.60)	
***Education level***					<0.001
Illiteracy or Primary school	1,299 (48.52)	776 (46.05)	373 (49.01)	150 (64.94)	
Junior middle School or above	1,378 (51.48)	909 (53.95)	388 (50.99)	81 (35.06)	
***Annual household income***					<0.001
Low (≤10,000 RMB)	785 (34.31)	416 (28.81)	271 (43.15)	98 (45.37)	
Moderate (10,001–30,000 RMB)	706 (30.86)	473 (32.76)	168 (26.75)	65 (30.09)	
High (>30,000 RMB)	797 (34.83)	555 (38.43)	189 (30.10)	53 (24.54)	
	***Healthy lifestyle indicators***				
***Smoking status***					<0.001
Lifetime non-smoker	2,070 (74.54)	1,429 (81.42)	472 (60.51)	169 (69.83)	
Former smoker	158 (5.69)	61 (3.48)	71 (9.10)	26 (10.74)	
Current smoker	549 (19.77)	265 (15.10)	237 (30.38)	47 (19.42)	
***Drinking status***					<0.001
Lifetime non-drinker	2345 (83.33)	1,536 (86.49)	595 (75.22)	214 (86.64)	
Former drinker	66 (2.35)	26 (1.46)	30 (3.79)	10 (4.05)	
Current drinker	403 (14.32)	214 (12.05)	166 (20.99)	23 (9.31)	
***Body mass index***, ***kg*** **/** ***m*** ^**2**^					0.364
Normal weight (18.5 ≤ BMI <24.0)	964 (34.94)	606 (34.75)	283 (36.42)	75 (31.51)	
Overweight (24.0 ≤ BMI <28.0)	1,163 (42.15)	742 (42.55)	313 (40.28)	108 (45.38)	
Obesity (BMI ≥ 28.0)	615 (22.29)	389 (22.31)	174 (22.39)	52 (21.85)	
Underweight (BMI < 18.5)	17 (0.62)	7 (0.40)	7 (0.90)	3 (1.26)	
	***Physical status and clinical parameters***				
***Presence of current or past disease***					<0.001
No	2,387 (84.83)	1,573 (88.57)	646 (81.67)	168 (68.02)	
Yes	427 (15.17)	203 (11.43)	145 (18.33)	79 (31.98)	
***FPG***					<0.001
FPG < 6.1 mmol/L	1,981 (92.96)	1,269 (94.63)	542 (92.33)	170 (83.74)	
6.1 ≤ FPG < 7.0 mmol/L	67 (3.14)	36 (2.68)	21 (3.58)	10 (4.93)	
FPG ≥ 7.0 mmol/L	83 (3.89)	36 (2.68)	24 (4.09)	23 (11.33)	
***TC***					0.026
TC < 5.2 mmol/L	1,860 (70.67)	1,206 (72.22)	510 (69.39)	144 (63.44)	
5.2 ≤ TC < 6.2 mmol/L	607 (23.06)	370 (22.16)	177 (24.08)	60 (26.43)	
TC ≥ 6.2 mmol/L	165 (6.27)	94 (5.63)	48 (6.53)	23 (10.13)	
***TG***					<0.001
TG < 1.7 mmol/L	1,840 (69.91)	1,197 (71.68)	511 (69.52)	132 (58.15)	
1.7 ≤ TG <2.3 mmol/L	411 (15.62)	244 (14.61)	120 (16.33)	47 (20.70)	
TG ≥ 2.3 mmol/L	381 (14.48)	229 (13.71)	104 (14.15)	48 (21.15)	
***LDL***					0.070
LDL < 3.4 mmol/L	2,279 (86.59)	1,458 (87.31)	638 (86.80)	183 (80.62)	
3.4 ≤ LDL <4.1 mmol/L	283 (10.75)	167 (10.00)	81 (11.02)	35 (15.42)	
LDL ≥ 4.1 mmol/L	70 (2.66)	45 (2.69)	16 (2.18)	9 (3.96)	
***HDL***					0.455
HDL ≥ 1.0 mmol/L	2,070 (74.54)	1,429 (81.42)	472 (60.51)	169 (69.83)	
HDL < 1.0 mmol/L	182 (6.91)	110 (6.59)	52 (7.07)	20 (8.81)	

Abbreviations: FPG, fasting plasma glucose; TC, total cholesterol; TG, triglycerides; HDL, high-density lipoprotein; LDL, low-density lipoprotein.

^a^Numbers do not add to total subjects due to missing data.

^b^ *P* values derived from chi-square tests.

### Distribution of SRH

More than half of the study population rated their health as “healthy” (63.1%), whereas 28.1% and 8.8% rated their health as “fair” or “unhealthy” (Table 1). Compared to males (47.7% of whom rated their health as “healthy”, 42.9% as “fair”, and 9.5% as “unhealthy”), females (72.5% of whom rated their health as “healthy”, 19.1% as “fair”, and 8.4% as “unhealthy”) had a higher likelihood of reporting a better SRH (OR = 0.29, 95% CI: 0.18–0.46) (Table 2). Health ratings declined with increasing age (*P* value for trend = 0.001) (Table 2).

**Table 2:** Cumulative univariate and multivariate analyses of factors associated with self-rated health among 2,814 adults from rural China, 2014.

Variables	Univariate		Multivariate^a^	
	OR	(95% CI)	OR	(95% CI)
***Age (years)***				
25–40	1.00	Ref.	1.00	Ref.
41–56	2.23	(1.95, 2.54)	1.93	(1.20, 3.09)
57–72	4.59	(3.39, 6.20)	3.00	(1.59, 5.66)
*P* value for trend^b^	<0.001		0.001	
***Gender***				
Male	1.00	Ref.	1.00	Ref.
Female	0.39	(0.25, 0.61)	0.29	(0.18, 0.46)
***Marital status***				
Never married, divorced or widowed	1.00	Ref.	1.00	Ref.
Married	0.50	(0.41, 0.61)	0.81	(0.70, 0.93)
***Number of family members living together***				
1–2	1.00	Ref.	1.00	Ref.
3–5	0.48	(0.42, 0.54)	1.18	(0.81, 1.71)
≥6	0.53	(0.46, 0.62)	1.08	(0.85, 1.37)
*P* value for trend^b^	<0.001		0.741	
***Type of employment***				
Farming	1.00	Ref.	1.00	Ref.
Non-farming	1.07	(0.81, 1.42)	0.77	(0.68, 0.88)
***Education level***				
Illiteracy or Primary school	1.00	Ref.	1.00	Ref.
Junior middle school or above	0.72	(0.64, 0.82)	0.73	(0.59, 0.91)
***Annual household income***				
Low (≤10,000 RMB)	1.00	Ref.	1.00	Ref.
Moderate (10,001–30,000 RMB)	0.57	(0.43, 0.76)	0.82	(0.62, 1.09)
High (>30,000 RMB)	0.50	(0.37, 0.67)	0.78	(0.57, 1.07)
*P* value for trend^b^	<0.001		0.128	
***Smoking status***				
Lifetime non-smoker	1.00	Ref.	1.00	Ref.
Former smoker	3.23	(2.27, 4.58)	1.20	(0.95, 1.51)
Current smoker	2.11	(1.31, 3.39)	1.02	(0.67, 1.55)
*P* value for trend^b^	0.001		0.978	
***Drinking status***				
Lifetime non-drinker	1.00	Ref.	1.00	Ref.
Former drinker	2.60	(1.98, 3.41)	0.99	(0.61, 1.60)
Current drinker	1.47	(1.10, 1.97)	0.82	(0.51, 1.33)
*P* value for trend^b^	0.003		0.429	
***Body mass index***, ***kg/m*** ^***2***^				
Normal weight (18.5 ≤ BMI < 24.0)	1.00	Ref.	1.00	Ref.
Overweight (24.0 ≤ BMI < 28.0)	0.99	(0.87, 1.12)	0.91	(0.77, 1.08)
Obesity (BMI ≥ 28.0)	1.00	(0.89, 1.11)	0.84	(0.68, 1.03)
Underweight (BMI < 18.5)	2.40	(1.29, 4.46)	1.90	(0.50, 7.25)
*P* value for trend^b^	0.357		0.173	
***Presence of current or past disease***				
No	1.00	Ref.	1.00	Ref.
Yes	2.30	(1.60, 3.32)	2.02	(1.66, 2.45)
***FPG***				
FPG < 6.1 mmol/L	1.00	Ref.	1.00	Ref.
6.1 ≤ FPG <7.0 mmol/L	1.59	(1.18, 2.16)	1.19	(0.62, 2.27)
FPG ≥ 7.0 mmol/L	2.84	(2.26, 3.56)	2.11	(1.31, 3.40)
*P* value for trend^b^	<0.001		0.019	
***TC***				
TC < 5.2 mmol/L	1.00	Ref.	1.00	Ref.
5.2 ≤ TC <6.2 mmol/L	1.20	(1.09, 1.31)	1.08	(0.90, 1.30)
TC ≥ 6.2 mmol/L	1.47	(1.12, 1.94)	1.54	(1.16, 2.06)
*P* value for trend^b^	<0.001		0.021	
***TG***				
TG < 1.7 mmol/L	1.00	Ref.	1.00	Ref.
1.7 ≤ TG <2.3 mmol/L	1.32	(1.02, 1.71)	1.20	(0.99, 1.45)
TG ≥ 2.3 mmol/L	1.31	(0.85, 2.01)	1.13	(0.66, 1.94)
*P* value for trend^b^	0.083		0.454	
***LDL***				
LDL < 3.4 mmol/L	1.00	Ref.	1.00	Ref.
3.4 ≤ LDL <4.1 mmol/L	1.29	(0.80, 2.07)	0.93	(0.48, 1.78)
LDL ≥ 4.1 mmol/L	1.06	(0.57, 2.01)	0.52	(0.20, 1.37)
*P* value for trend^b^	0.100		0.125	
***HDL***				
HDL ≥ 1.0 mmol/L	1.00	Ref.	1.00	Ref.
HDL < 1.0 mmol/L	1.17	(0.89, 1.54)	0.83	(0.55, 1.27)

Abbreviations: OR, odds ratio; CI, confidence interval; FPG, fasting plasma glucose; TC, total cholesterol; TG, triglycerides; HDL, high-density lipoprotein; LDL, low-density lipoprotein.

^a^All variables were included in cumulative multivariate analyses.

^b^ *P* values for trend were calculated by cumulative univariate and multivariate analyses, treating categorical variables as continuous variables.

### Risk factor analysis

In multivariate analyses, married status (Adjusted OR = 0.81, 95% CI = 0.70–0.93, married *vs*. never married, divorced or widowed), non-farming (Adjusted OR = 0.77, 95% CI = 0.68–0.88, non-farming *vs*. farming) and possession of higher levels of education (Adjusted OR = 0.73, 95% CI = 0.59–0.91, junior middle school or above *vs*. illiteracy or primary school) increased the likelihood of reporting a better SRH (Table 2). Subjects with a history of disease had worse SRH (Adjusted OR = 2.02, 95% CI = 1.66–2.45, with current or past disease *vs*. without current or past disease). The association with SRH increased significantly with the abnormal levels of FPG (*P* value for trend = 0.019) and was markedly elevated among individuals with a high level of FPG (Adjusted OR = 2.11, 95% CI = 1.31–3.40, FPG ≥ 7.0 mmol/L *vs*. FPG < 6.1 mmol/L). However, there was no such statistical association observed for the borderline high level of FPG and SRH (Adjusted OR = 1.19, 95% CI = 0.62–2.27, 6.1 ≤ FPG < 7.0 mmol/L *vs*. FPG < 6.1 mmol/L) after adjustments. Similarly, elevated level of TC was statistically correlated with poorer SRH (*P* value for trend = 0.021). Categorized by TC level, the high level of TC increased the likelihood of reporting a worse SRH (Adjusted OR = 1.54, 95% CI = 1.16–2.06, TC ≥ 6.2 mmol/L *vs*. TC < 5.2 mmol/L), however, there was no such statistical association found for the borderline high level of TC and SRH (Adjusted OR = 1.08, 95% CI = 0.90–1.30, 5.2 ≤ TC < 6.2 mmol/L *vs*. TC < 5.2 mmol/L). Abnormality of TG, LDL, and HDL showed no statistical correlation with SRH.

The multiplicative interactions between age, gender and other explanatory variables were analyzed respectively. Except for annual household income, none of the other variables showed significant interaction with gender when other explanatory variables were controlled. Therefore, only the effect of annual household income on SRH stratified by gender was explored (Table 3). In multivariate analyses, there was a clear gradient correlation between annual household income and SRH in males. That is, the higher the annual household income, the better SRH (*P* value for trend = 0.018; Adjusted OR = 0.36, 95% CI = 0.19–0.71, high (>30,000 RMB) *vs*. low (≤10,000 RMB)). However, there was no such independent association in females (Table 3).

**Table 3:** Cumulative univariate and multivariate analyses of the association between household annual income and self-rated health among 2,814 adults (gender-stratified models) from rural China, 2014.

Variables	Male					Female				
	No. (%)	Univariate		Multivariate^b^		No. (%)	Univariate		Multivariate^b^	
		OR	(95% CI)	OR	(95% CI)		OR	(95% CI)	OR	(95% CI)
***Annual household income***										
Low (≤10,000 RMB)	329 (41.02)	1.00	Ref.	1.00	Ref.	456 (30.69)	1.00	Ref.	1.00	Ref.
Moderate (10,001–30,000 RMB)	230 (28.68)	0.58	(0.30, 1.14)	0.76	(0.43, 1.34)	476 (32.03)	0.64	(0.39, 1.03)	0.81	(0.53, 1.25)
High (>30,000 RMB)	234 (30.30)	0.36	(0.19, 0.71)	0.55	(0.34, 0.88)	554 (37.28)	0.68	(0.49, 0.94)	0.87	(0.50, 1.51)
*P* value for trend^a^		0.005		0.018			0.023		0.662	

Abbreviations: OR, odds ratio; CI, confidence interval.

^a^ *P* values for trend were calculated by cumulative univariate and multivariate analyses, treating the annual household income as continuous variable.

^b^All variables including demographic characteristics, socioeconomic characteristics, healthy lifestyle indicators, physical status and clinical parameters were included in cumulative multivariate analyses.

### Spousal SRH concordance

As shown in Table 4, of 697 couples in which both partners provided SRH data, there was a statistically significant spousal concordance for SRH, although the degree of concordance was low (Weighted kappa = 0.09, *P* = 0.001). Spearman’s rho test also showed that the SRH of one partner was mildly but significantly correlated with that of the other partner (Spearman’s rho = 0.12, *P* = 0.001).

**Table 4:** Concordance between respondents’ SRH and spouses’ SRH among 697 couples from rural China, 2014^a^.

Respondents’ SRH	Spouses’ SRH			Total
	Healthy	Fair	Unhealthy	
*Healthy*	263	42	30	335 (48.06%)
*Fair*	207	65	27	299 (42.90%)
*Unhealthy*	38	17	8	63 (9.04%)
*Total*	508 (72.88%)	124 (17.79%)	65 (9.33%)	697

Abbreviations: SRH, self-rated health.

^a^Weighted Kappa was calculated to evaluate the concordance between respondents’ SRH and spouses’ SRH. To identify correlation between respondents’ SRH and spouses’ SRH, chi-square analysis and Spearman’s rank test was used. Weighted Kappa = 0.09, *P* = 0.001; Spearman’s rho = 0.12, *P* = 0.001; chi-square [4] = 15.02, *P* = 0.005.

## Discussion

To our knowledge, this is the first study assessing both the determinants and spousal concordance for SRH for general population in rural China. Of the male and female respondents, 9.5% and 8.4% reported “unhealthy” SRH in rural China. Poor SRH was positively correlated with disease status and abnormality of some clinical parameters but not with presence of unhealthy lifestyle indicators including smoking, drinking, and obesity. High household income was predictive of better SRH among males but not among females. Among couples, a statistically significant positive spousal concordance for SRH was observed, although the concordance strength was low. These findings will be useful to formulate appropriate strategies for health intervention.

Our study showed that 8.8% of respondents rated their health status as “unhealthy”, while more than 60% reported “healthy” SRH. These proportions were in the middle of the wide range reported by prior studies from Asian countries^11, 15, 16, 23, 24^. The variation found among populations may largely explain the heterogeneity of proportions across studies. It is well known that advancing age and low education levels are positively associated with poor SRH^10, 11^. Our results further confirmed this correlation. With regard to gender, reports of its association with SRH have not been fully consistent^25–27^. In this study, SRH in women overall appeared to be better compared with SRH in men. Since poorer SRH reflects a higher burden of diseases^28^, gender disparity in SRH ratings could be partially explained by the fact that men in this population were more likely to report having a disease history (Male *vs*. Female: 31.4% *vs*. 13.2%, driven by a higher prevalence of upper digestive tract disorders, respiratory disease, and cardiovascular illness; data not shown).

It has been suggested previously that high household income is predictive of better SRH^9^. Notably, in this study, an independent association between annual household income and SRH was found in males but not in females. This gender disparity in the relationship of family income and health has also been observed by other researchers^23, 24^. It could be explained partially by specific sociological factors. For instance, household income is largely dependent on men’s income in rural China, which may affect their control over family resources, access to health care and decision-making power^29^. Accordingly, the impact of income inequality (e.g., low-income households *vs*. high-income households) on health is more likely to be observed in men (major controllers) than in women. However, the mechanism by which income inequality is linked to health and SRH yet to be convincingly established.

Consistent with most previous studies, current or past disease was strongly associated with poorer SRH^2, 28^. For clinical parameters, the degree of association increased with the abnormal levels of FPG and TC even after adjusting for disease status and other potential confounders. Additionally, high levels of FPG (≥7.0 mmol/L) and TC (≥6.2 mmol/L) were significantly associated with poorer SRH. The correlations between biochemical indexes and SRH observed by us and others groups may be the result of physical sensations associated with disease progression, such as fatigue and poor sleep patterns, indicating that SRH has a biologic basis and it may serve as a barometer of physiologic states^13, 14^. For borderline high levels of FPG and TC as well as abnormality of TG, LDL and HDL, their association with SRH were not statistically significant after adjustments. This can be partially explained by the fact that rural residents rarely underwent biochemical examinations and most were unaware of the abnormality of clinical parameters. Thus, in contrast to high levels of clinical indexes which may influence health perception and relate to SRH ratings, marginally elevated levels of biochemical indicators without accompanying symptoms and signs would be unlikely to have an impact on SRH reporting^13, 14^. For unhealthy lifestyle indicators (such as smoking, drinking, and obesity), a null association was observed, in contrast with results from economically developed regions^30^. Taken together these findings may reflect the fact that adults in rural China often lack basic knowledge about disease prevention and are thus not aware that risk factor exposure may have adverse health effects^31^. In support of this assumption, other studies found that obesity is not regarded as unhealthy, but rather as a matter of good fortune in Chinese culture especially in rural area. This is especially true in rural areas, where it is believed that only rich people could afford to eat more and gain weight^32^. In view of the fact that physical health but not unhealthy lifestyles which are regarded as major causes of modern-day disease^33^, plays an important role in SRH in rural China, more measures for health promotion and intervention are recommended for rural individuals to improve lifestyle and risk perception. Further research is needed to confirm our findings.

Our study also augments knowledge regarding spousal SRH concordance in heterosexual couples based on a large sample. As expected, we found that the SRH of one partner was significantly associated with that of the other partner, although the degree of spousal concordance was low. This phenomenon may arise from the combined effect of shared and non-shared factors. The contribution of sharing a similar environment, social network, and financial resources (including specific factors such as age, education level and annual household income) to similarity in spousal SRH could be attenuated to some extent by effects of non-shared factors (including smoking status, BMI and history of disease) for respondents and their partners (Supplementary Table S1). Moreover, differing genetic characteristics in couples could also reduce the degree of concordance. Thus, life features shared in couples could lead to the observed significant though low SRH concordance. This finding may serve as a first step toward developing couple-based interventions to jointly improve SRH for both partners.

This study has several limitations. First, the possibility of a selection bias (e.g. bias introduced by exclusion of individuals aged below 25 and above 69) may undermine the generalizability of our findings to a wider population. Second, bias potentially introduced by the individuals who did not participate must be noted, as such bias may have blurred the age/gender-related association. Third, with the exception of clinical parameters, all information was self-reported data. Recall bias or under-reporting of risk factors cannot be excluded, although interviews were administered by well-trained interviewers in a one-on-one private setting. Fourth, our findings may be subject to potential endogeneity bias. Additionally, information about depression, health service utilization and social networks was not collected, and their association with SRH could thus not be evaluated. Finally, owing to its cross-sectional nature, the temporal order of observed associations with SRH cannot be established.

In conclusion, this study adds to current understanding of self-rated health and its determinants. SRH differed significantly with socio-demographic characteristics, disease status, and clinical parameters, but did not vary with healthy lifestyle indicators in rural China. Among couples, the shared lifestyle features may lead to a significant although low SRH concordance. These findings will be useful for future improvement in subjective well-being and objective health in economically developing regions.

## Electronic supplementary material

Supplementary Table S1

## References

[1] Lam CL, Tse EY, Gandek B (2005). Is the standard SF-12 health survey valid and equivalent for a Chinese population?. Qual Life Res.

[2] Fayers PM, Sprangers MA (2002). Understanding self-rated health. Lancet.

[3] Idler EL, Benyamini Y (1997). Self-rated health and mortality: a review of twenty-seven community studies. Journal of health and social behavior.

[4] Waller G, Janlert U, Norberg M, Lundqvist R, Forssen A (2015). Self-rated health and standard risk factors for myocardial infarction: a cohort study. BMJ open.

[5] Lundberg O, Manderbacka K (1996). Assessing reliability of a measure of self-rated health. Scand J Soc Med.

[6] DeSalvo KB, Bloser N, Reynolds K, He J, Muntner P (2006). Mortality prediction with a single general self-rated health question. A meta-analysis. J Gen Intern Med.

[7] Wu S (2013). The relationship between self-rated health and objective health status: a population-based study. BMC Public Health.

[8] Damian J, Pastor-Barriuso R, Valderrama-Gama E (2008). Factors associated with self-rated health in older people living in institutions. BMC Geriatr.

[9] Kondo N (2009). Income inequality, mortality, and self rated health: meta-analysis of multilevel studies. BMJ.

[10] Stanojevic Jerkovic, O., Sauliune, S., Sumskas, L., Birt, C. & Kersnik, J. Determinants of self-rated health in elderly populations in urban areas in Slovenia, Lithuania and UK: findings of the EURO-URHIS 2 survey. *Eur J Public Health* (2015). 10.1093/eurpub/ckv097 26163468

[11] Haseli-Mashhadi N (2009). Self-Rated Health in middle-aged and elderly Chinese: distribution, determinants and associations with cardio-metabolic risk factors. BMC Public Health.

[12] Hanibuchi T, Nakaya T, Murata C (2012). Socio-economic status and self-rated health in East Asia: a comparison of China, Japan, South Korea and Taiwan. Eur J Public Health.

[13] Jylha M, Volpato S, Guralnik JM (2006). Self-rated health showed a graded association with frequently used biomarkers in a large population sample. J Clin Epidemiol.

[14] Saudny, H., Cao, Z. & Egeland, G. M. Poor self-reported health and its association with biomarkers among Canadian Inuit. *International journal of circumpolar health* **71** (2012). 10.3402/ijch.v71i0.18589 PMC3426798 22973568

[15] Mao Z, Wu B (2007). Urban-rural, age and gender differences in health behaviours in the Chinese population: findings from a survey in Hubei, China. Public Health.

[16] Shen C (2014). Self-rated health and mortality in a prospective Chinese elderly cohort study in Hong Kong. Prev Med.

[17] Kim ES, Chopik WJ, Smith J (2014). Are people healthier if their partners are more optimistic? The dyadic effect of optimism on health among older adults. J Psychosom Res.

[18] Brown DC, Hummer RA, Hayward MD (2014). The Importance of Spousal Education for the Self-Rated Health of Married Adults in the United States. Population research and policy review.

[19] Liu F (2012). The anyang esophageal cancer cohort study: study design, implementation of fieldwork, and use of computer-aided survey system. PLoS One.

[20] Zhou, B. F. & Cooperative Meta-Analysis Group of the Working Group on Obesity in, C. Predictive values of body mass index and waist circumference for risk factors of certain related diseases in Chinese adults–study on optimal cut-off points of body mass index and waist circumference in Chinese adults. *Biomed Environ Sci* **15**, 83–96 (2002). 12046553

[21] Zhu J, Gao R (2016). Guidelines for prevention and treatment of dyslipidemia in Chinese adults (revised edition). Chinese Circulation Journal.

[22] Glucose tolerance and mortality: comparison of WHO and American Diabetes Association diagnostic criteria. The DECODE study group. European Diabetes Epidemiology Group. Diabetes Epidemiology: Collaborative analysis Of Diagnostic criteria in Europe. *Lancet* **354**, 617–621 (1999). 10466661

[23] Lim WY, Ma S, Heng D, Bhalla V, Chew SK (2007). Gender, ethnicity, health behaviour & self-rated health in Singapore. BMC Public Health.

[24] Yamazaki S, Fukuhara S, Suzukamo Y (2005). Household income is strongly associated with health-related quality of life among Japanese men but not women. Public Health.

[25] Rohlfsen LS, Jacobs Kronenfeld J (2014). Gender Differences in Trajectories of Self-Rated Health in Middle and Old Age: An Examination of Differential Exposure and Differential Vulnerability. Journal of aging and health.

[26] Jylha M, Guralnik JM, Ferrucci L, Jokela J, Heikkinen E (1998). Is self-rated health comparable across cultures and genders? *The journals of gerontology*. Series B, Psychological sciences and social sciences.

[27] Xu J, Zhang J, Feng L, Qiu J (2010). Self-rated health of population in Southern China: association with socio-demographic characteristics measured with multiple-item self-rated health measurement scale. BMC Public Health.

[28] Malmusi D, Artazcoz L, Benach J, Borrell C (2012). Perception or real illness? How chronic conditions contribute to gender inequalities in self-rated health. Eur J Public Health.

[29] Wu J (2004). Education-related gender differences in health in rural China. Am J Public Health.

[30] Sodergren M, Sundquist J, Johansson SE, Sundquist K (2008). Physical activity, exercise and self-rated health: a population-based study from Sweden. BMC Public Health.

[31] Xiaohui H (2008). Urban-rural disparity of overweight, hypertension, undiagnosed hypertension, and untreated hypertension in China. Asia-Pacific journal of public health/Asia-Pacific Academic Consortium for Public Health.

[32] Li ZB, Ho SY, Chan WM (2004). Obesity and depressive symptoms in Chinese elderly. Int J Geriatr Psychiatry.

[33] He J (2005). Major causes of death among men and women in China. N Engl J Med.

