# Supplementary material for: Epidemiology of self-rated health in rural China: a population-based cross-sectional study
Source: Sci Rep. 2017 Jun 30;7:4459. doi: 10.1038/s41598-017-04381-6 (PMC5493681; doi:10.1038/s41598-017-04381-6)
Supplement: Supplementary file 1 — Supplementary Table S1 [file 41598_2017_4381_MOESM1_ESM.doc]

**Epidemiology of self-rated health in rural China: a population-based cross-sectional study**

Fangfang Liu, Chaoting Zhang, Yongmei Liang, Qiuju Deng, Dong Hang, Yaqi Pan, Xiang Li, Zhonghu He, Mengfei Liu, Ying Liu, Jingjing Li, Tao Ning, Chuanhai Guo, Ruiping Xu, Lixin Zhang, Hong Cai and Yang Ke

| **Supplementary Table S1. Socio-demographic characteristics and associated factors with SRH for 697 couples in rural China, 2014** | | | | |
| --- | --- | --- | --- | --- |
| ***Variables***^a^ | ***Husbands, No. (%)*** | ***Wives, No. (%)*** | ***Correlation***^b^ | ***P* value**^c^ |
| ***Age (years)*** |  |  | 0.87*** | < 0.001 |
| Median (IQR) | 50 (44-61) | 50 (44-60) |  |  |
| 25-40 | 89 (12.77) | 88 (12.63) |  |  |
| 41-56 | 343(49.21) | 368 (52.80) |  |  |
| 57-69 | 265 (38.02) | 241 (34.58) |  |  |
| ***Type of employment*** |  |  | 0.15*** | < 0.001 |
| Farming | 312 (45.15) | 598 (86.54) |  |  |
| No farming | 379 (54.85) | 93 (13.46) |  |  |
| ***Education level*** |  |  | NA | NA |
| Illiteracy or primary school | 660 (100.00) | 660 (100.00) |  |  |
| Junior middle school or above | 0 (0.00) | 0 (0.00) |  |  |
| ***Annual household income*** |  |  | 0.37 | < 0.001 |
| Low (≤ 10,000 RMB) | 195 (35.85) | 203 (32.95) |  |  |
| Moderate (10,001-30,000 RMB) | 163 (29.96) | 197 (31.98) |  |  |
| High (> 30,000 RMB) | 186 (34.19) | 216 (35.06) |  |  |
| ***Smoking status*** |  |  | 0.004 | 0.097 |
| Lifetime non-smoker | 226 (32.71) | 688 (99.57) |  |  |
| Former smoker | 105 (15.20) | 0 (0) |  |  |
| Current smoker | 360 (52.10) | 3 (0.43) |  |  |
| ***Drinking status*** |  |  | 0.05 | 0.168 |
| Lifetime non-drinker | 380 (54.52) | 690 (99.00) |  |  |
| Former drinker | 44 (6.31) | 0 (0) |  |  |
| Current drinker | 273 (39.17) | 7 (1.00) |  |  |
| ***Body mass index, kg/m^2^*** |  |  | -0.01 | 0.832 |
| Normal weight (18.5 ≤ BMI < 24.0) | 214 (31.24) | 235 (34.31) |  |  |
| Overweight (24.0 ≤ BMI < 28.0) | 298 (43.50) | 291 (42.48) |  |  |
| Obesity (BMI ≥ 28.0) | 170 (24.82) | 157 (22.92) |  |  |
| Underweight (BMI < 18.5) | 3 (0.44) | 2 (0.29) |  |  |
| ***Presence of current or past disease*** |  |  | 0.11** | 0.005 |
| No | 594 (85.22) | 578 (82.93) |  |  |
| Yes | 103 (14.78) | 119 (17.07) |  |  |
| ***FPG*** |  |  | 0.04 | 0.168 |
| FPG < 6.1 mmol/L | 483 (94.71) | 539 (92.45) |  |  |
| 6.1≤ FPG < 7.0 mmol/L | 11 (2.16) | 18 (3.09) |  |  |
| FPG ≥ 7.0 mmol/L | 16 (3.14) | 26 (4.46) |  |  |
| ***TC*** |  |  | 0.05 | 0.357 |
| TC <5.2 mmol/L | 487 (73.68) | 443 (67.02) |  |  |
| 5.2 ≤ TC < 6.2 mmol/L | 128 (19.36) | 163 (24.66) |  |  |
| TC ≥ 6.2mmol/L | 46 (6.96) | 55 (8.32) |  |  |
| ***TG*** |  |  | -0.01 | 0.871 |
| TG < 1.7 mmol/L | 429 (64.90) | 459 (69.44) |  |  |
| 1.7 ≤ TG < 2.3 mmol/L | 113 (17.10) | 110 (16.64) |  |  |
| TG ≥ 2.3 mmol/L | 119 (18.00) | 92 (13.92) |  |  |
| ***LDL*** |  |  | 0.02 | 0.233 |
| LDL < 3.4 mmol/L | 578 (87.44) | 558 (84.42) |  |  |
| 3.4 ≤ LDL < 4.1 mmol/L | 67 (10.14) | 82 (12.41) |  |  |
| LDL ≥ 4.1 mmol/L | 16 (2.42) | 21 (3.18) |  |  |
| ***HDL*** |  |  | 0.04 | 0.329 |
| HDL ≥ 1.0 mmol/L | 596 (90.17) | 632 (95.61) |  |  |
| HDL <1.0 mmol/L | 65 (9.83) | 29 (4.39) |  |  |
| Abbreviations: FPG, fasting plasma glucose; TC, total cholesterol; TG, triglycerides; HDL, high-density lipoprotein; LDL, low-density lipoprotein; NA, not applicable. | | | | |
| ^a^Numbers do not add to total subjects (697) due to missing data. | | |  |  |
| ^b^Correlation refers to Spearman's rho. | | | | |
| ^c^*P* values were derived from Fisher’s exact tests (two-tailed). | | | | |
| **P* < 0.05; ** *P* < 0.01; *** *P* < 0.001. | |  |  |  |
